# Occurrence of Mycotoxins in Dried Fruits Worldwide, with a Focus on Aflatoxins and Ochratoxin A: A Review

**DOI:** 10.3390/toxins15090576

**Published:** 2023-09-18

**Authors:** Miguel Ángel González-Curbelo, Bulent Kabak

**Affiliations:** 1 Departamento de Ciencias Básicas, Facultad de Ingeniería, Universidad EAN, Calle 79 no 11-45, Bogotá 110221, Colombia; 2 Department of Food Engineering, Faculty of Engineering, Hitit University, Corum 19030, Turkey; 3 Biotechnology Laboratory, Machinery and Manufacturing Technology Application and Research Center, Hitit University, Corum 19030, Turkey

**Keywords:** drying methods supply chain, monitoring, food safety, climate change, storage conditions, exposure

## Abstract

Dried fruits are popular and nutritious snacks consumed worldwide due to their long shelf life and concentrated nutrient content. However, fruits can be contaminated with various toxigenic fungal species during different stages, including cultivation, harvesting, processing, drying, and storage. Consequently, these products may contain high levels of mycotoxins. This risk is particularly pronounced in developed countries due to the impact of climate change. Several factors contribute to mycotoxin production, including the type of fruit, geographical location, climate conditions, harvest treatments, and storage management practices. The main mycotoxins in dried fruits are aflatoxins (AFs) and ochratoxin A (OTA), which can induce human health problems and economic losses. Mycotoxin contamination can vary significantly depending on the geographic origin of dried fruits (vine fruits, figs, dates, apricots, prunes, and mulberries). The aim of this review was to fill the knowledge gap by consolidating data from various regions to understand the global picture and identify regions with higher contamination risks. By consolidating research from various origins and stages of the supply chain, the review intends to shed light on potential contamination events during pre-harvest, drying, storage, and trading, while also highlighting the effects of storage conditions and climate change on mycotoxin contamination.

## 1. Introduction

Dried fruits are enjoyed by populations worldwide as a shelf-stable alternative to fresh fruit, overcoming common barriers that prevent people from consuming fruit [1]. Dried fruits are a good source of dietary fibre, minerals, vitamins, and bioactive compounds and antioxidants, which play a protective role in human health. They also have a low to moderate glycemic index [2].

The world’s dried fruit production has shown a positive trend over the last decade, reaching a total amount of 3.1 million metric tons in the 2021/2022 season. Among the dried fruits, dried vine fruits (raisins, sultanas, and currants) continued to be the most produced by volume, accounting for over 1.3 million metric tons, representing 43% of the total share. Dates followed with a total production of over 1.1 million metric tons, accounting for over 36% and experiencing the most significant upward trend over the last decade, with an average yearly increase of about 47 metric tons from 2012/2013 to 2021/2022. Prunes (6%), dried apricots (5%), dried figs (5%), and sweetened dried cranberries (5%) accounted for the remaining 21%. The United States (15%) and Turkey (15%) have been the global supply leaders over the last five years, together accounting for 30% of the world´s dried fruit production. Iran ranked third in dried fruit production with 12%, followed by Saudi Arabia (7%) and China (6%). In terms of global estimated consumption, the Middle East, Europe, and Asia are the top three dried fruit-consuming regions, with 32%, 23%, and 20% of the world share, respectively [3].

**Figure 1:**
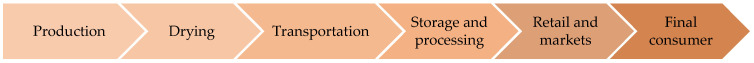
The major components of the supply chain of dried fruits.

The supply chain of dried fruits (Figure 1) involves several key stages, including fruit harvesting, washing and sorting, drying, storage, and processing. The specific details of the supply chain may vary depending on the type of dried fruit (e.g., raisins, figs, date) and the location where it is produced. Throughout this supply chain, factors, like climate, drying, and storage conditions, play vital roles in determining the quality of the final product.

Fruits can be dried using traditional methods under the sun for several days, through solar drying, or artificially in dryers for a shorter period. Drying under the sun and solar drying differ in their approach and control over the drying process. Drying under the sun, also known as sun drying, relies on the sun’s heat and energy to remove moisture from the substance. In this method, the material to be dried is spread out in direct sunlight, typically on mats, racks, or the ground. However, this process lacks precise control and can be affected by external factors, such as weather conditions, humidity, and temperature fluctuations. Consequently, drying times can vary significantly, and there is a risk of contamination from dust, insects, or other environmental factors. On the other hand, solar drying is a more controlled and efficient method that harnesses solar energy while incorporating specific design features to optimize the drying process. It involves the use of solar dryers, which are devices designed to capture the sun’s energy and create an environment conducive to drying. These solar dryers typically consist of an enclosed structure with transparent surfaces (such as glass or plastic) that allow sunlight to enter while trapping the heat inside. As a result, solar drying creates a controlled drying environment, protecting the material from dust, insects, and other contaminants. By carefully regulating the drying parameters, solar drying can achieve faster and more consistent results compared to sun drying [4].

Dried fruits with low moisture content are more resistant to undesirable microorganisms, such as bacteria, yeasts, and molds, compared to fresh fruits. Water activity (a_w_) plays an important role in determining the quality change and microbial growth or survival [5]. Dried fruits with an a_w_ value of ≤0.60 are microbiologically stable. However, the microorganisms that survive during the drying process remain dormant for a long period and become active once fruits are rehydrated [6]. Dried fruits are susceptible to invasion by xerophilic fungi, such as *Aspergillus flavus*. *Aspergilli* belonging to section *Flavi* can grow and produce mycotoxins down to 0.73 and 0.85 a_w_, respectively [7].

Mycotoxins are toxic compounds that are naturally produced by several field and storage fungi. They can cause adverse health effects in humans and animals, ranging from acute poisoning to long-term effects, such as immune deficiency and certain cancers. The severity of these effects depends on various factors, including the type of mycotoxin, its dose, genetic predisposition, and physiological factors. Mycotoxigenic fungi are typically not host-specific, but their occurrence is mainly associated with specific crops, depending on the growing area and climatic conditions. A single fungus can produce different mycotoxins, leading to concurrent contamination of food with multiple mycotoxins [8,9]. These mycotoxins enter the food chain when crops become infected either before or after harvest, especially under humid and warm conditions. They are commonly found in certain foodstuffs, such as cereals, oilseeds, spices, cocoa and coffee beans, nuts, and dried fruits. Notably, mycotoxins are heat-stable compounds, which means they can survive traditional heat treatments, like baking and roasting. As a result, they can also be detected in processed foods and may contaminate animal products, such as milk, meat, and eggs, through infected feed [10,11]. Currently, numerous published reviews reported the occurrence of mycotoxin contamination in various foodstuffs [12,13,14,15,16]. These reports indicated that mycotoxin contamination in agricultural products is considered a global issue, particularly in the case of tropical and subtropical countries.

**Figure 2:**
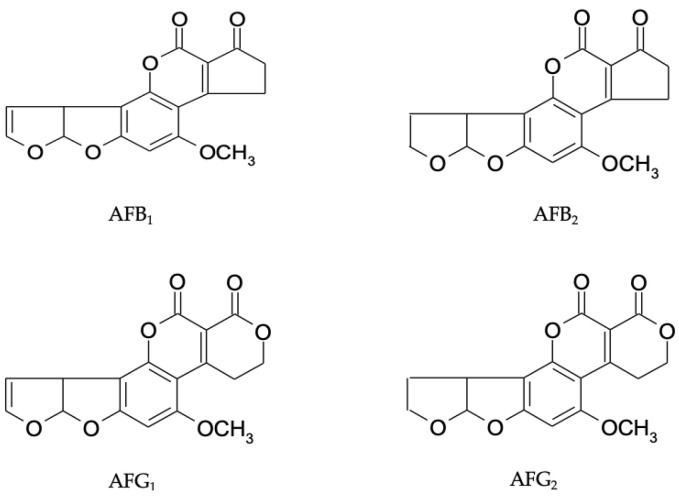
Chemical structures of AFB_1_, AFB_2_, AFG_1_, and AFG_2_.

Aflatoxins (AFs) and ochratoxin A (OTA) are among the main mycotoxins that contaminate foods worldwide. The fungi *A. flavus* and *Aspergillus parasiticus* are common contaminants of dried fruits, capable of producing genotoxic carcinogens known as AFs. *A. flavus* mainly produces aflatoxin B_1_ (AFB_1_) and aflatoxin B_2_ (AFB_2_), whereas *A. parasiticus* produces AFB_1_, AFB_2_, aflatoxin G_1_ (AFG_1_) and aflatoxin G_2_ (AFG_2_). AFB_1_ is the most frequently found aflatoxin in contaminated crops, and the others three are generally not found in the absence of AFB_1_ [10,17]. AFB_1_ has been classified as a human carcinogen (Group 1) by the International Agency for Research on Cancer (IARC) [18]. AFs pose various toxic effects on animals and humans, including mutagenicity, teratogenicity, and immunotoxicity [19]. The European Commission has established maximum levels (MLs) of 2 and 4 μg/kg for AFB_1_ and the sum of AFs (AFT, AFB_1_ + AFB_2_ + AFG_1_ + AFG_2_), respectively, in dried fruits intended for human consumption [20]. The chemical structures of naturally occurring AFs are shown in Figure 2.

**Figure 3:**
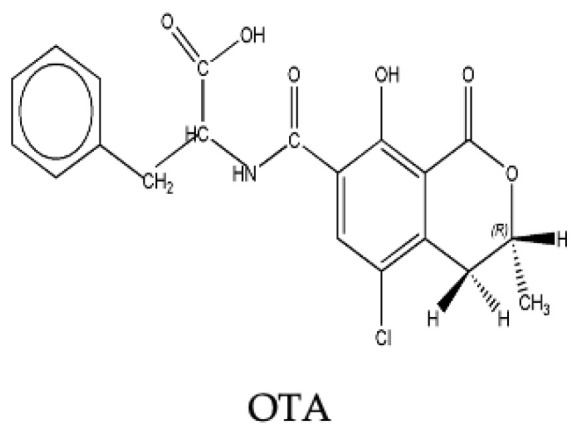
Chemical structure of OTA.

OTA (Figure 3) is the second most frequently reported mycotoxin in dried fruits. It is synthesized by several species of *Aspergillus* and *Penicillium*, particularly *Aspergillus carbonarius*, *Aspergillus ochraceus*, *Aspergillus westerdijkiae*, and *Penicillium verrucosum* [21,22]. OTA is known to exert several adverse health effects, particularly on the kidney and urinary tract, and has been associated with fatal human kidney disease, referred to as Balkan endemic nephropathy [23]. OTA has demonstrated neurotoxic, immunotoxic, and teratogenic effects at higher doses in mammalian species, while its genotoxic potential is controversial [11]. The IARC classified OTA as “possibly carcinogenic to humans” (Group 2B) based on sufficient evidence of carcinogenicity in animal studies and inadequate evidence in humans [18]. Moreover, a combined intake of different types of mycotoxins, like AFs and OTA, may lead to an additive or synergistic threat and to more pronounced health effects [24,25]. It has been shown that OTA could increase the mutagenicity of AFB_1_ in the case of their co-occurrence in the foodstuffs [26]. Currently, in the European Union (EU), MLs of 8 and 2 μg/kg have been set for OTA in dried vine fruit and dried figs, and other dried fruits, respectively [27]. Several reviews have focused on OTA contamination in plant-derived [13,28,29] and animal-derived food products [29]. Other mycotoxins, such as fumonisins, especially fumonisin B_2_ (FB_2_) [30,31,32], T-2 toxin [33,34], HT-2 toxin [35], enniatins (Enns) [34,35], and diacetoxyscirpenol (DAS) [35], have been rarely reported in dried fruits worldwide.

Methods used for the analysis of mycotoxins vary widely. The reference methods for the determination of AFs [36] and OTA [37] in dried fruits and other foodstuffs using high-performance liquid chromatography coupled with fluorescence detection (HPLC-FLD) are available. The analysis involves immunoaffinity clean-up prior to HPLC-FLD determination, which can effectively improve the signal-to-noise ratio and increase the sensitivity and accuracy of the detection method. The multi-toxin methods based on liquid chromatography coupled with tandem–mass spectrometry (LC-MS/MS) have also been popular methods over the last decade for the quantitative determination of hundreds of mycotoxins together with other chemical hazards, such as plant toxins, pesticides, and veterinary drugs in the same analysis. LC-MS/MS methods have the advantages that no sample clean-up and post-column derivatization are needed. However, multi-toxin methods often suffer from lower accuracy and recoveries compared to single-analyte methods, due to the analytical compromise adopted to cover a wide spectrum of different compounds [10]. Apart from chromatographic methods, immuno-based analyses, such as enzyme-linked immunosorbent assay (ELISA), dipsticks, and biosensors are also available for the determination of mycotoxins with minor relevance [11,13,38,39].

Climate change has emerged as a significant threat to food safety, as it can lead to alterations in temperature and humidity, which in turn can significantly impact the growth of toxigenic fungi and the formation of mycotoxins in dried fruits. In addition, elevated temperatures can stimulate the expression of genes involved in mycotoxin biosynthesis, potentially leading to increased mycotoxin production. Moreover, climate change may also impact the geographic distribution of mycotoxin-producing fungi, resulting in new areas of contamination.

This review aims to describe the profiles of mycotoxins in dried fruits (vine fruits, figs, dates, plums, apricots, mulberries, and other fruits) worldwide and to explore the impact of storage conditions and climate change on fungal colonization and subsequent mycotoxin formation. By consolidating occurrence data on mycotoxins in dried fruits from various geographic regions, this review aims to provide a comprehensive understanding of the global picture and identify regions with higher and increasing contamination risks.

## 2. Mycotoxin Profile in Dried Fruits

### 2.1. Dried Vine Fruits

Fungal invasion occurs at the point of berry injury caused by insect or bird feeding, mechanical or growth cracks, or lesions resulting from powdery mildew infection or *esca* (black measles) berry damage, which results in cracking [40]. In warmer growing areas, *Alternaria*, *Botrytis, Cladosporium*, and *Rhizopus* species can attack during veraison, which represents the transition from berry growth to berry ripening, while *Aspergillus*, *Penicillium,* and *Eurotium* are more common in sun-dried grapes. More specifically, *Aspergillus* section *Nigri* species, particularly *A. carbonarius*, which is resistant to germicidal UV light and strong sunlight heating, can often infect berries during both sun-drying and storage, resulting in OTA formation [41]. In a study by Magnolia et al. [42], *A. niger* var *niger*, *A. niger* var *awamori*, and *A. carbonarius* were found to be predominant species in black and white dried vine fruits from the Mendoza and San Juan provinces of Argentina. Similarly, black Aspergilli (*A. niger* aggregate and *A. carbonarius*) were the prevalent species in raisins from western regions of Greece [31]. Of the 400 *Aspergillus* strains isolated from raisin samples (*n* = 40) from four distinct raisin vineyards located in Fresno, California, only 12 isolates (3%) identified as *A. carbonarius* were found to produce OTA [43]. A mycological survey of dried grapes from Turkey showed the incidence of four genera: *Aspergillus*, *Penicillium*, *Trichoderma*, and *Cladosporium*. Among the species, *A. niger* was predominant (69 out of 96 isolates, 72%), and 59.4% of them were capable of OTA production [44].

Inappropriate storage conditions, such as warm temperature, high humidity, and improper/insufficient ventilation of the silos, also affect the growth of toxigenic fungi and subsequently mycotoxin synthesis [45]. The European Commission has set an ML of 8 μg/kg for OTA in dried vine fruit (currant, raisin, and sultanas) [27].

OTA is the most frequently notified mycotoxin in dried vine fruits in the Rapid Alert System for Food and Feed (RASFF) database. In the interval of years from 2017 to 2021, a total of 143 notifications involving OTA were published for dried grapes. Three countries were the main players in the notification for the country of origin. Turkey was the leading country of origin with 77 notifications (53.8%), followed by Iran (13.3%, *n* = 19) and Uzbekistan (11.9%, *n* = 17). A large majority of notifications regarding dried grapes between 2017 and 2021 were classified as “border rejection notifications” (59.4%), followed by “alert notifications” (23.1%) and “information notifications” (17.5%). 

Between 2017 and 2021, mycotoxins were responsible for the majority of the notifications (74.8%, *n* = 107) in dried grapes. The notifications on mycotoxins all concerned OTA reported in dried grapes by various countries of origin, except for only one notification on AFs. Sulfite was the second reason for notifications on dried grapes (11.2%, *n* = 16) from various countries. Compared to 2018 and 2019, there was a decreasing trend in the notification of OTA in dried grapes for the last two consecutive years (2020–2021). In 2021, there were seven notifications concerning OTA in dried grapes, five of which (71.4%) were from Uzbekistan, one from Pakistan, and one from Turkey. The concentrations of OTA reported in dried grapes varied from 16.5 μg/kg (from Uzbekistan) to 99.8 μg/kg (Pakistan) [46].

The occurrence data on OTA in dried grapes worldwide during the last three decades are summarised in Table 1. In the surveys conducted from 1998 to date in 17 countries, OTA occurred in 2450 out of 3117 dried grape samples (78.6%). Among 17 countries, both the incidence (100%) and the highest dried grape contamination (138.3 μg/kg) reported were in Greece.

Data presented in Table 1 and RASFF reports show that OTA contamination in dried grapes is a significant problem, especially in Turkey, Iran, Uzbekistan, Pakistan, Greece, and South Africa. A survey carried out to quantify OTA contamination in dried grapes from various geographical origins, including Chile, China, Iran, Turkey, South Africa, and the United States, showed that the highest average contamination was measured in samples from Turkey (3.1 μg/kg) [59].

Data are also available about OTA contamination in dried grapes consumed in European countries. A total of 562 results on OTA occurrence in dried grapes were provided by 4 countries (France, Germany, Greece, and the United Kingdom). The prevalence rate of OTA contamination in dried grapes was 46.2% in France, 94.3% in Germany, 54.9% in Greece, and 91.1% in the United Kingdom, with levels up to 4.3, 21.4, 16.5, and 53.6 μg/kg, respectively [77].

In the food group “dried fruits”, dried grapes made the largest contribution to OTA exposure in Turkish adults. The mean and 95th percentile (P95) exposure estimates to OTA through the consumption of dried grapes were 0.006 and 0.028 ng/kg bw/day, respectively. Despite dried grapes having the highest mean and P95 OTA concentrations among the samples, which included dried figs, cereals and cereal-based products, nuts (pistachios and hazelnuts), coffee, chocolate, chili, wine, and beer, they made only a minor contribution to OTA exposure (1.6–4%) [75]. In the EU-coordinated program, the mean dietary exposure to OTA through the consumption of dried grapes for adults varied across different countries: it ranged from 0.02 to 0.03 ng/kg bw/day in Finland, 0.06 to 0.14 ng/kg bw/day in France, 0.001 to 0.002 ng/kg bw/day in Greece, and 0.06 ng/kg bw/day in the United Kingdom [77].

AF contamination was rarely reported in dried grapes. In an earlier study, dried vine fruits sold in the United Kingdom were observed to be free of AFs [76]. However, 4 out of the 20 raisin samples (20%) from Morocco had AFs up to a level of 13.9 μg/kg [78]. In Greece, 23% of the dried vine fruit samples contained AFB_1_ with a mean concentration of 0.15 μg/kg [54].

The data on AF contamination in dried grapes mainly come from Iran and Pakistan. In 2009–2011, AFB_1_ was detected in only 1 out of 22 raisin samples (0.64 μg/kg) in Iran [56]. In another study in Iran, an AF incidence of 18.8% was recorded for 16 raisin samples, with a concentration range of 0.85–2.14 μg/kg [79]. In Pakistan, Asghar et al. [62] examined 170 raisin samples during three consecutive years (2012–2014) and reported that 3 out of 53, 2 out of 51, and 3 out of 66 raisin samples contained AFs at levels 0.15–1.64, 0.22–1.54, and 0.17–2.23 μg/kg, respectively. In a limited survey in Pakistan conducted by Luttfullah and Hussain [80], AF incidence was 20% (two out of 10 samples) in raisins, with a mean value of 5.05 μg/kg. In another study by Iqbal et al. [63], AFs were detected in 6 out of 17 raisin samples (35.3%) up to a concentration of 13.5 μg/kg (mean = 5.1 μg/kg). In another limited survey from Pakistan conducted in 2013, 9 out of 21 raisin samples (42.9%) were found to contain AFs with a maximum concentration of 13.45 μg/kg [81].

The species that produce fumonisins, belonging to the genus *Fusarium*, most frequently *Fusarium verticillioides* and *Fusarium proliferatum*, have been often isolated from various cereals, predominantly in maize [9,82]. Further research also showed that FB_2_ as well as fumonisin B_4_ (FB_4_) are synthesized along with OTA in various products other than cereals [83], such as coffee beans and grapes, by certain strains of *Aspergillus* section Nigri [31,33,84,85,86]. In a study by Susca et al. [86], 48 strains of *Aspergillus* section *Nigri* isolated from grapes and raisins were examined for the presence of the fumonisin biosynthetic gene *fum8* in relation to FB_2_. The *fum8* gene was recorded in 11 *A. niger* strains, and 9 of them were found to produce FB_2_. In another study, four out of 66 *A. niger* strains were found to produce FB_2_ and FB_4_ in raisins [33].

More recently, all four strains isolated from dried grapes sold in the Slovak food market belonging to *A. niger* were found to produce fumonisin B_1_ (FB_1_) and FB_2_, while other *Aspergillus* section *Nigri* isolates, including *Aspergillus welwitschiae*, *A. carbonarius*, and *Aspergillus tubingensis*, were not capable of producing fumonisins [66]. In Greece, FB_2_ was found in 12 out of 42 raisin samples (29%) at levels of 7.1–25.5 μg/kg, 6 of which co-occurred with OTA [31]. In another study, contamination of Turkish dried grapes with FB_2_ was only recorded in 1 out of 60 samples (1.7%) at a level of 8.8 μg/kg [74]. Other mycotoxins, such as T-2 toxin (10.2 μg/kg), AFG_2_ (4.2 μg/kg), and enniatinB (EnnB) (21.4 μg/kg) in raisins from Valencia, Spain, were also rarely reported [34]. 

### 2.2. Dried Figs

Fig fruits (*Ficus carica* L.) are highly susceptible to physical damage and may be infested with mold spores at all stages, starting from the ripening process on the tree branch to harvesting, processing, and storage [87]. The most important postharvest fungal-related diseases of figs are smut (*A. niger*), *Alternaria* rot (*Alternaria tenuis* and others), *Botrytis* rot (*B. cinerea*), fig endosepsis (*Fusarium moniliforme*, *Fusarium solani*), and *Aspergillus* rot (*A. flavus*, *A. parasiticus*) [88,89]. 

The main hazards in the dried fig trade are AFs and OTA. Before the drying process, figs are fumigated, and those that give a bright greenish-yellow color (BGYF) are removed from the lot under 360 nm ultraviolet (UV) light in the processing plant [87]. Although this method is effective and practical in removing figs containing AFs from the batches, it can produce false positive and negative results. This technique sorts only figs that give BGYF on the outer skin of the fruit due to kojic acid. However, a critical issue is that AFs biosynthesis can occur inside the fruit cavity, infected with aflatoxigenic molds from the *ostiole*, especially during ripening, and it is not possible to separate these figs from the lot with the current system. Moreover, this application can only be used for AF-contaminated figs, and it cannot separate OTA or other mycotoxin-contaminated figs. Regulated synthetic plant protection products, such as aluminum phosphite and magnesium phosphite, can be used to prevent mold growth in fig processing plants, but these fumigants may also have adverse effects on the environment and human health.

In the interval of years from 2017 to 2021, a total of 325 RASFF notifications concerning dried figs were published. AFs were the source of 82.8% (269 notifications) of these notifications, while OTA was in second place with 34 (10.5%) notifications. The remaining 22 notifications (6.7%) are related to other hazards (insects, sulfites, pesticides, and missing documents) [46]. Many surveys have been conducted worldwide concerning the incidence of AFs in dried figs. Table 2 summarizes the results of studies within the last decade.

In the surveys conducted worldwide from 2002 to the present, AFB_1_ was found in 2902 out of 25,842 dried fig samples (11%), with levels reaching up to 696.3 μg/kg. In a recent study by Kabak [98], the mean dietary exposure estimates in Turkish consumers of dried figs ranged from 0.003 to 0.004 ng/kg bw/day for AFB_1_ and from 0.0004 to 0.006 ng/kg bw/day for AFT. Dried figs made only a minor contribution to AFB_1_ and AFT exposure (1%), likely due to low dried fig consumption.

On the contrary, little information is available about OTA contamination and exposure estimates. In an earlier study, Pavón et al. [99] reported that OTA incidence in dried figs from Spain was 54.3% (*n* = 35) with a maximum concentration of 245.3 μg/kg. In a United States study on dried figs, OTA was detected in only 4 out of 88 samples, with levels ranging from 0.5 to 3.3 μg/kg [43]. In Iran, almost half of the dried fig samples (*n* = 22) contained OTA with a maximum concentration of 10 μg/kg [92]. Later, Kulahi and Kabak [75] reported that seven dried fig samples originating from Turkey were contaminated with OTA, with a maximum concentration of 1.55 μg/kg. This divergence was explained by the fact that the distribution of toxins is heterogeneous from one batch to another and varies from year to year depending on climate variations.

### 2.3. Dates

In date fruits (*Phoenix dactylifera* L.), studies have shown the involvement of pathological disorders and mycotoxin-producing fungi, mainly *A. flavus*, *A. parasiticus*, and *A. niger*. The growth of toxigenic fungi on dates, under favorable climatic conditions, such as high humidity and temperature, can result in the formation of various mycotoxins, mainly AFs, and OTA. Table 3 summarizes the occurrence and concentrations of AFs and OTA in dates marketed in various countries.

In total, 16 date varieties at 3 stages of maturation collected from the 1999 harvests in the United Arab Emirates (UAE) were monitored for AFs, sterigmatocystin, and fungal counts. Aflatoxigenic *A. flavus* isolates were identified in 10 out of 16 date varieties at the first stage of maturation. However, after adverse storage conditions for 14 days, all samples were free from both AFs and sterigmatocystin [104]. On the contrary, Ahmed et al. [105] indicated that *A. parasiticus* was able to penetrate intact date fruits from the UAE and synthesize AFs after incubation at 28 °C for 10 days in all ripening stages, except the final stage (*Tamr*), which did not support mold growth.

In another survey conducted in Saudi Arabia by Gherbawy et al. [106], 24 fungal species belonging to 12 genera were isolated from retail date fruits (*n* = 50). The prevalent specied in date fruits were *A. flavus*, *A. niger*, *Penicillium chrysogenum*, and *Rhizopus stolonifera*. Among these species, 7 out of 8 *A. flavus* isolates and 9 out of 36 *A. niger* isolates were capable of producing AFs and OTA, respectively. In Yemen, AFs were monitored in dates by thin-layer chromatography (TLC) and were found in 2 out of 20 samples (10%) at high concentrations (110 and 180 μg/kg). The study also indicated widespread contamination of dates by the *Aspergillus* genus, especially *A. niger* [103]. Aidoo et al. [107] identified *A. flavus* and *A. parasiticus* in some imported date fruits in the United Kingdom. 

In a study from Pakistan [80], two date samples (*n* = 20) were found to contain AFs at levels of 2.1 and 2.9 μg/kg. However, in a later study also from Pakistan, AFs were detected in 38 out of 96 date samples (39.6%) and 18 out of 57 date products (date cookies, date cake, and date halva) collected from retail stores in southern areas of the Punjab and Khyber Pakhtunkhwa regions. The maximum concentrations recorded were 26.60 μg/kg (mean = 4.11 μg/kg) in dates and 16.70 μg/kg (mean = 3.65 μg/kg) in date products. The EU MLs of 2 and 4 μg/kg for AFB_1_ and AFT were exceeded in 16 and 20 date samples, respectively [101]. Similarly, out of the 15 dried dates sold in the northern and Khyber Pakhtunkhwa regions of Pakistan, nine samples were positive for AFs, with mean levels of 4.50 μg/kg for AFB_1_ and 6.32 μg/kg for AFT [81]. In another survey from 2012–2015, AFs were recorded in 25 out of 170 date samples (15%) from Pakistan, with concentrations ranging from 0.24 to 5.87 μg/kg [102].

In an Iranian study on dried fruits collected from retail stores in Hamadan city, AFs were detected in 9 out of 22 date samples (40.9%) with concentrations ranging from 0.9 to 8.1 μg/kg (mean = 2.6 μg/kg). In addition, 22.7% of the date samples contained OTA, and two of them showed the co-occurrence of AFB_1_ and OTA. The levels of OTA ranged from 0.5 to 2.1 μg/kg, with a mean value of 1.2 μg/kg [92]. In the study of Rahimi and Shakerian [58], OTA was found in only 2 out of 10 dates collected from Iran, at levels of 1.4 and 3.6 μg/kg.

Similarly, Iamanaka et al. [47] reported the presence of OTA in two out of 20 dried dates samples imported and sold in Brazil. In Shanghai, China, 16 mycotoxins were monitored in 40 date fruit samples, and only OTA was found in 9 samples (22.5%) with a maximum concentration of 61.4 μg/kg [100].

The available studies also showed that date fruits can be contaminated with mycotoxins other than AFs and OTA. In Egypt, 28 date samples were collected in 2016 and analyzed for the presence of 295 fungal and bacterial metabolites. A total of 30 toxic fungal metabolites were determined in date samples. Among these fungal metabolites, four types of ochratoxin, namely OTA (11%), ochratoxin B (11%), ochratoxin C (4%), and ochratoxin alpha (7%) were detected. The concentration ranges for OTA and ochratoxin B in three positive date samples were 1.48–6070 and 0.28–692 μg/kg, respectively. In addition, the co-occurrence of FB_2_ with four types of ochratoxins (A, B, C, and alpha) was observed in date samples, indicating fungal attack by *A. niger* species during storage. Two date samples (7%) were positive for FB_2_, with levels of 4.99 and 16.2 μg/kg, while only one sample contained AFB_1_ (14.4 μg/kg) and AFB_2_ (2.44 μg/kg). However, kojic acid, which occurred in 43% of the date samples, had the highest maximum concentration (90,400 μg/kg) among fungal metabolites [32].

Azaiez et al. [35] reported 16 mycotoxin results for 75 date samples collected from Tunisian (*n* = 48) and Spanish (*n* = 27) markets. Eight mycotoxins, namely EnnB, enniatinB_1_ (Enn B_1_), enniatinA_1_ (EnnA_1_), AFB_2_, AFG_1_, AFG_2_, OTA, and DAS, were recorded in date samples from Tunis. However, only three mycotoxins, namely EnnB, EnnB1, and EnnA1, were found in dates from Spain, with incidence rates of 66.7%, 7.4%, and 44.4%, respectively. EnnB was the prevalent mycotoxin (68%) in date samples with the reported maximum concentration of 190 μg/kg. In addition, EnnA_1_ co-occurred with EnnB in 26 samples, 3 of which also contained EnnB_1_. While AFB_1_ was not recorded in any date samples, AFB_2_, AFG_1_, and AFG_2_ were detected in 33.3%, 16.7%, and 31.3% of date samples from Spain with concentrations up to 1.3, 1.8, and 2.2 μg/kg, respectively. Out of the 48 date samples from the Tunisian market, 38% were contaminated with OTA, with levels ranging from 0.57 to 3.34 μg/kg (mean = 1.26 μg/kg); seven of them co-occurred with AFs. In addition, two date samples from Spain contained DAS, but the levels were below the limit of quantification (LOQ).

Between 2017 and 2021, 13 notifications on dates were transmitted through the RASFF system. Among these, six were classified as information notifications, five as border rejections, and two as alert notifications. For 31% (*n* = 4) of these notifications, Tunisia was the country of origin, followed by Pakistan (23%, *n* = 3) and Egypt (23%, *n* = 3). AFs were the most recurrent issues, with five notifications, followed by insects (*n*= 4), pesticides (chlorpyrifos (*n* = 2), fenpyroximate (*n* = 1)), OTA (*n* = 1), and molds (*n* = 1). In a recent notification sent by Belgium in 2021, a case of too high content of co-occurrence of AFs (AFB_1_ = 70 μg/kg, AFT = 73 μg/kg) and OTA (47 μg/kg) in dates from Tunisia was reported [46].

### 2.4. Dried Plums (Prunes)

Plums (*Prunus domestica*) and prunes, like other stone fruits, are very susceptible to postharvest diseases, such as blue mold rot (*Penicillium expansum*), brown rot (*Monilia laxa*, *Monilia fructicola*), gray rot (*B. cinerea*), *Mucor* rot (*Mucor piriformis*), and *Rhizopus* rot (*Rhizopus* spp.) [108]. Mycotoxin-producing fungi and mycotoxins have also been recorded in prunes worldwide. Table 4 summarizes the occurrence and concentrations of AFs and OTA in prunes marketed in various countries.

OTA is reported to be the main mycotoxin problem in prunes [111]. In an earlier study by Zohri and Abdel-Gawad [109], three samples of dried plums from Egypt were monitored for fungal microflora as well as mycotoxins (AFs, OTA, T-2 toxin, zearalenone, patulin, citrinin, sterigmatocystin, and DAS) content by TLC. Dried plums were found to be contaminated with various fungi, especially those belonging to the genera *Penicillium*, *Aspergillus*, *Cladosporium*, *Alternaria,* and *Eurotium*. Among the targeted mycotoxins, only OTA was detected in all three dried plum samples at very high levels of 210–280 μg/kg. A lower level of OTA was determined in 83.9% of imported prune samples in Germany, with a maximum concentration of 0.07 μg/kg [112].

The most abundant species isolated from dried plum samples from Brazil was *A. niger*, with an infection rate of 8%. However, only 1 of the 21 dried plum samples was found to be positive for OTA [47]. In Iran, 2 and 3 out of 15 prune samples contained AFB_1_ and OTA with levels of 0.13–1.17 μg/kg and 0.22–2.62 μg/kg, respectively [110].

A higher incidence (38%) of AFs in dried plums (8 out of 21 samples) from Pakistan was recorded, with contamination levels varying from 0.04 to 7.45 μg/kg (mean = 2.42 μg/kg) for AFB_1_ and from 0.04 to 14.76 μg/kg (mean = 3.72 μg/kg) for AFT [81]. In another survey from Pakistan, 3 out of 10 dried plum samples (30%) were found to be contaminated with AFs with levels of 2.36–7.41 μg/kg [102]. Similarly, 6 out of 16 plum samples (37.5%) from Pakistan had AFs up to a level of 8.5 μg/kg [63].

Dried plums (*n* = 27) and other dried fruits (dried vine fruits, dried figs, dates, and dried apricots) were collected in 2012–2013 from different markets in Tunisia and Valencia, Spain, and tested for 16 mycotoxins. Dried plums had the lowest contamination rate (25.9%) for the targeted mycotoxins among all the dried fruit samples tested. This is explained by the use of additives, such as potassium sorbate and sorbic acid in some prune samples sold in Valencian markets. Nevertheless, prune samples among other dried fruits had the highest levels of AFG_2_ (15.6 μg/kg), HT-2 toxin (10 μg/kg), and DAS (135 μg/kg). This study also indicated that AFG_2_, OTA, and HT-2 toxin co-occurred in seven dried plum samples, four of them purchased from Tunisia and three from Spain [35]. While AFs and OTA were not determined in prune samples, all four prune samples from Turkey were positive for fumonisins, ranging from 670 to 1684 μg/kg [30].

No notifications were reported on mycotoxins in prunes, while there were only seven RASFF notifications on prunes from 2017 to 2021, four of which were concerned with food additives, namely sorbic acid [46].

### 2.5. Dried Apricots

Dried apricot (*Prunus armeniaca* L.) has a moderate to low risk of mycotoxins accumulation during pre- and post-harvest stages. According to RASFF reports, OTA was notified only once in dried apricots from Turkey in the last few years [46]. Table 5 summarizes the occurrence and concentrations of AFs and OTA in dried apricots marketed in various countries.

The treatment of apricots with sulfur dioxide has been reported to prevent fungal contamination. Neither AFs nor OTA were detected in sulfured and naturally dried apricot samples from different processing plants in Turkey [114]. However, in the study of Gunsen and Buyukyoruk [113], 3 out of 15 analyzed dried apricot samples from Turkey were AFB_1_ positive, with a mean concentration of 1.44 μg/kg. Later, Bircan [72] showed that only 1 out of 20 dried apricot samples intended for export contained OTA at a concentration of 0.97 μg/kg. In a two-year study conducted in Turkey, AFs, and OTA were detected in 2 and 6 out of 26 dried apricot samples at levels of up to 10.5 and 34.4 μg/kg, respectively. Apart from AFs and OTA, 45.5% of samples had fumonisins up to a level of 1492 μg/kg [30].

In Pakistan, AFs were found in 4 out of 20 dried apricot samples (20%) at levels varying from 1.5 to 10.3 μg/kg, with a mean concentration of 4.55 μg/kg [80]. In a later study in Pakistan, 7 out of 20 dried apricot samples were positive for AFs (limit of detection (LOD) = 0.04 μg/kg). The mean and maximum concentrations in dried apricots were 3.93 and 7.15 μg/kg for AFB_1_ and 4.75 and 11.50 μg/kg for AFT, respectively [81]. In the four-year study of Asghar et al. [102], 21 of the 65 dried apricot samples (32%) from Pakistan were positive for AFs with a range of concentrations of 0.31–11.1 μg/kg. In another study in Pakistan, 3 out of 13 dried apricot samples were contaminated with AFs, with a maximum value of 11.85 μg/kg [63].

In Iran, dried apricot was found to be the most frequently contaminated material with AFs among the dried fruits (dried figs, mulberry, and date), with an incidence of 81.8%. While eight dried apricot samples had AFB_1_ above the EU ML of 2 μg/kg, five samples exceeded the EU ML of 4 μg/kg for AFT [92]. The concentrations of AFB_1_ and AFT in dried apricot samples varied from 0.4 to 7.1 μg/kg and from 0.4 to 8.5 μg/kg, respectively. In another Iranian study, 9 of the 30 apricot samples contained AFB_1_, with levels ranging from 0.21 to 5.33 μg/kg. For OTA, only one apricot sample was positive, with a level of 2.83 μg/kg [110]. Similarly, Rahimi and Shakerian [58] reported that only 1 out of 15 apricot samples from Iran had OTA, with a concentration of 2.8 μg/kg.

A higher level of OTA within the range of 50 and 110 μg/kg was recorded by the TLC technique in all three dried apricot samples sold in Egypt, whereas the samples were found to be negative for other mycotoxins, AFs, T-2 toxin, zearalenone, patulin, citrinin, sterigmatocystin, and DAS [109]. However, none of the dried apricot samples (*n* = 20) from Brazil were found to be positive for OTA [47].

In addition to AFs and OTA, other mycotoxins, such as Enns and T-2 toxin, may be found in dried apricots. Of the 27 dried apricot samples purchased from supermarkets in Tunisia (*n* = 12) and Spain (*n* = 15), more than half of the samples (59.3%) contained at least 1 of the 16 targeted mycotoxins at quantifiable concentrations. In the apricots, EnnB was the most frequently detected compound, with an incidence rate of 40.7% and with a mean level of 133 μg/kg, followed by HT-2 toxin (29.6%, maximum concentration = 5.39 μg/kg), EnnA_1_ (14.8%, 5.7 μg/kg), EnnB_1_ (11.1%, 57.1 μg/kg), OTA (11.1%, 0.3 μg/kg), and AFG_2_ (11.1%, < LOQ) [35].

### 2.6. Dried Mulberries

Surveys on mycotoxin contamination in dried mulberries (*Morus alba* L., *Morus rubra* R.) are less frequent compared with other dried fruits. In a study by Luttfullah and Hussain [80], four out of 15 dried mulberry samples sold in Pakistan were found to be positive for AFs with a mean concentration of 2.22 μg/kg (concentration range 1.0–3.5 μg/kg). In a later study by Asghar et al. [102], two out of 10 dried mulberry samples contained AFs with levels of 1.36 and 2.22 μg/kg. 

In Iran, 10 out of 22 (45.5%) dried mulberry samples were positive for AFs and OTA. In these samples, AFB_1_, AFT, and OTA concentrations varied from 0.35 to 8.4 μg/kg (mean = 3.0 μg/kg), 0.6 to 11.8 μg/kg (mean = 4.1 μg/kg), and from 0.4 to 3.4 μg kg^−1^ (mean = 1.75 μg/kg), respectively [92]. However, in the study by Kaya and Tosun [30] in Turkey, no AFs were determined in any of the six dried mulberry samples. In that study, one out of six dried mulberry samples contained OTA and fumonisins with concentrations of 3.96 and 1372.5 μg/kg, respectively. In the first half of 2021, the Netherlands reported three instances of AFs presence in dried mulberries from Turkey, two of which also contained OTA [46].

### 2.7. Other Dried Fruits

Mycotoxin contamination in other fruits, such as cranberries, goji fruit, and jujube, has been rarely reported. All six dried cranberry samples from Poland, the United States, and Canada were free from AFs and OTA. None of the five goji fruit samples from China had AFB_1_, whereas three of them contained OTA with levels of 1.11–2.08 μg/kg [65]. For dried jujube from Beijing, China, all samples (*n* = 20) from the year 2013 were contaminated with OTA, but at very low concentrations, with a maximum of 0.18 μg/kg [115].

## 3. Effect of Storage Conditions on Mycotoxin Formation

The humidity and temperature in the storage are key environmental factors affecting fungal growth and mycotoxin production [116], making them the main reasons for mycotoxin problems in dried fruits. High temperatures and elevated humidity levels create favorable conditions for toxigenic fungal growth, which increases the risk of mycotoxin contamination in dried fruits and other commodities [117]. The shelf life of dried fruits can be prolonged if they are dried to a water activity value at which molds, yeasts, and bacteria cannot grow (a_w_ < 0.65). Therefore, it is essential to store dried fruits in cool and dry environments to minimize the growth of fungi and reduce the likelihood of mycotoxin formation. Additionally, it is crucial to ensure that the storage area is dry and well-ventilated to prevent moisture condensation on the fruits. If further hot spots form where temperature and moisture increase, secondary mycotoxin formation may occur. For this reason, any possible source that increases humidity in the dried fruits or the surrounding environment must be eliminated. Direct contact of dried fruit containers with floors or walls needs to be prevented by placing a palette or a similar separator [87].

The utilization of hermetic storage in the implementation of the dry chain offers a preventive measure against mold growth during the storage process, thereby reducing food losses and minimizing the risk of exposure to mycotoxins. While the dry chain has traditionally proven highly effective in arid climates with naturally low ambient relative humidity levels that prevent spoilage, its effectiveness becomes compromised in tropical regions with elevated humidity and temperatures. In such settings, the drying process becomes more challenging, and conventional storage methods involving porous fabric bags expose dried foods to moisture absorption, leading to spoilage caused by mold growth and contamination by mycotoxins. It is important to note that low temperatures merely decelerate mold growth in inadequately dried products. Moreover, the prevalent high humidity within cold storage facilities may result in additional water absorption by food items, increasing their susceptibility to mold growth once they are reintroduced to higher temperatures, such as during power outages, packaging, or transport [118]. However, Naeem et al. [119] observed that the cold storage of dried figs was the most effective way to prevent mycotoxin formation when compared to open-air storage and hermetic (air-tight sealed) storage in glass jars. In another report, it is suggested that dried figs should be stored and retailed at refrigeration temperatures (<10 °C) to avoid the formation of AFs by *A. flavus* [120].

Pests, such as insects and rodents, can damage dried fruits and create entry points for fungal contamination in storage conditions. Additionally, some pests can carry fungal spores on their bodies, contributing to mycotoxin contamination. Implementing effective pest control measures in the storage area can help prevent pest infestations and reduce the risk of mycotoxin contamination in dried fruits. Adhering to good manufacturing practices during the processing and storage of dried fruits is crucial for minimizing mycotoxin contamination. This includes maintaining cleanliness, regularly inspecting and cleaning storage areas and equipment, and following proper hygiene practices. Proper handling and storage techniques can help prevent cross-contamination and minimize the risk of mycotoxin development [87].

## 4. Impact of Climate Change on Mycotoxin Contamination

Environmental factors are the primary driving forces behind the patterns of fungal attack and mycotoxin formation in agricultural products. The emergence of new climatic conditions may induce changes in the dynamics of fungal attacks and mycotoxin formation. The industrial revolution has contributed to the growing amount of pollutants in the atmosphere, such as fossil fuel emissions from factories and greenhouse gas emissions. The accumulation of these gases in the atmosphere is the leading cause of global warming and climate change [121].

The concentration of CO_2_ in the atmosphere reached 416 ppm in 2021 [122] and is estimated to increase (double or triple) within the near future, resulting in global warming of 2–5 °C, depending on industrialization and human activity [123]. Global warming may cause certain crops to mature and ripen earlier in certain regions, thereby altering harvesting, drying, and storage practices. With the global climate changes, there is also likely to be an increase in the incidence of droughts, excessive precipitation, and flooding, leading to a decrease in plant resilience and yields, a deterioration in crop quality, and an increase in insects and other pest populations, distribution, and attacks [123,124].

The European Food Safety Authority (EFSA) has stated that certain regions, such as northern Europe, will be positively affected, while others will suffer detrimental effects due to anticipated environmental changes [125]. The Mediterranean region, in particular, has been identified as highly vulnerable to climate change, with warming occurring at a rate 20% faster than the global average. Furthermore, the Mediterranean region is expected to experience an increase in drought frequency and intensity, a decrease in precipitation in the eastern Mediterranean, and a temperature increase of 2–3 °C. The number of hot days with temperatures exceeding 30 °C is also projected to increase in several countries, including Spain, Morocco, Algeria, central Italy, the Balkans, and central Turkey [121]. This could increase the frequency of *Aspergillus* species and AF contamination in the region.

High levels of atmospheric CO_2_ can also contribute to AF contamination. Many studies have reported that the crop cultivation environment is expected to undergo significant changes due to the projected doubling or tripling of CO_2_ concentrations, from 350 ppm to a range of 700–1000 ppm. Medina et al. demonstrated that AFB_1_ production was stimulated in climate change scenarios related to elevated CO_2_ levels, especially when coupled with drought stress. Although increased CO_2_ levels did not affect the growth of *Aspergillus* species, the study revealed a relative increase in the structural aflD and regulatory aflR genes, indicating a significant impact on the biosynthetic pathway involved in AF production, particularly at an elevated temperature of 37 °C and under water stress conditions [126].

Recent predictions indicate that pests and diseases are migrating to the poles at a rate of 3–5 km/year on a global scale, and the diversity of pest populations will also change significantly, having profound economic implications for staple food production systems. A recent study of wheat diseases and climatic change suggests that the physiology of wheat is modified when exposed to elevated CO_2_, resulting in increased severity of diseases, such as *Septoria tritici* blotch (STB) and *Fusarium Head Blight* (FHB) [127]. However, few studies have examined the impact of three-way interactions between temperature, water availability, and CO_2_ on growth and mycotoxin production by toxigenic species [123].

It can be broadly stated that mycotoxin risk from climate change will likely be highest in developed countries with temperate climates, such as in Europe and the United States. As temperatures in these regions warm to 33 °C, close to the optimal temperature for AF production, the risk of mycotoxin contamination will increase. This risk will be amplified if crops susceptible to AFs, such as peanuts and maize, are grown more frequently to take advantage of the changing climate. The fact that AFs are among the most dangerous mycotoxins further exacerbates this risk compared to other climatic regions. Thus, even in areas where AFs have not been a significant problem in the past, they may become a substantial risk [128,129].

On the other hand, countries with very cold climates, such as Norway, Canada, and Russia, may not see significantly greater concerns from AFs than already exist, as even global warming will not result in temperatures optimal for *A. flavus* growth. In hot tropical climates, other concerns may take precedence if temperatures increase at the same rate. It is possible that fungal growth and mycotoxin production could be reduced in extreme temperatures above 40 °C, leading to the extinction of fungi that thrive in high temperatures. However, if temperatures do not become much higher and drought conditions become more frequent, this may stimulate AF contamination [128].

It has been reported that fruit crops are particularly vulnerable to climate change as compared to field crops due to their extended flowering period. Alterations in climatic parameters have had a significant influence on the growth and development of fruit crops, resulting in changes in flowering patterns, modifications in fruit quality, and shifts in disease incidence [130]. 

Climatic conditions strongly influence the presence of OTA in grapes [131]. It has been demonstrated that the growth rate of *A. carbonarius* and its production of OTA were higher under simulated temperature conditions (18/31 °C) compared to climate change scenarios (20/37 °C) [132,133]. Correspondingly, there was an overall upregulation of genes involved in OTA biosynthesis at 18/31 °C, which coincided with increased mycotoxin production. Additionally, Oueslati et al. [134] revealed that *A. carbonarius* strains isolated from Tunisian grapes exhibited significantly enhanced growth at 20/30 °C compared to 20/37 °C, while growth was even slower at 25/42 °C. Garcia-Cela et al. [135] also observed a reduction in the growth rates of *A. ochraceus* and *A. carbonarius*, as well as OTA production, following an increase in temperature. Based on these findings, an anticipated increase in temperatures may lead to a decrease in *A. carbonarius* and OTA production in grapes.

Climate change poses a significant and multifaceted challenge to the drying process and dried fruit supply chain, particularly in the context of AFs and OTA contamination. The choice of drying method can vary depending on factors, such as climate, tradition, and available resources. In many Mediterranean countries, including Turkey, Greece, and Tunisia, sun drying is a commonly used method for drying fruits due to their favorable climate conditions [111,136,137,138,139]. Conversely, countries with less sunshine, or those with a more industrialized approach to fruit processing, may prefer artificial dryers. Therefore, it is challenging to provide a specific breakdown by country, as the choice of drying method can be influenced by multiple factors. Rising temperatures and changing precipitation patterns can lead to altered drying conditions. Increased temperatures may expedite the drying process, potentially reducing the available timeframe for proper drying and decreasing the risk of mycotoxin contamination. However, elevated humidity levels associated with climate change can create a more conducive environment for fungal growth during drying and storage. Additionally, erratic rainfall patterns may disrupt traditional drying methods, forcing producers to rely more on mechanical drying. Thus, climate change can necessitate adjustments in traditional practices to mitigate the increased risk of mycotoxin contamination.

## 5. Conclusions

This review highlights the significant threat posed by mycotoxins in dried fruits, with a particular focus on AFs and OTA. While AFs and OTA represent major concerns, it is worth noting that other mycotoxins, like Enns, FB_2_, and T-2/HT-2 toxins, have been infrequently reported in various dried fruits. Notably, dried fruits primarily sourced from Iran, Pakistan, and Turkey exhibit a higher likelihood of mycotoxin contamination compared to those from other regions. Furthermore, the concentration of mycotoxins in dried fruits varies from year to year, closely tied to climate fluctuations. To minimize the impact of climate change on mycotoxin synthesis in dried fruits, a multifaceted approach that includes both mitigation and adaptation strategies is required. This involves implementing rigorous storage conditions, including precise control of temperature, humidity, moisture levels, packaging, and pest management, all while adhering to good manufacturing practices. Notably, it is crucial to acknowledge that, apart from dried vine fruits, many non-European countries lack established MLs for OTA in dried fruits, allowing companies to introduce their products into domestic markets without adequate oversight. Given the potential health risks, particularly for toddlers and young children, there is an urgent need to establish MLs for OTA in dried figs, dates, dried apricots, prunes, and dried mulberries.

## Figures and Tables

**Table 1:** Summary of the OTA occurrence data in dried vine fruits marketed in various countries.

Country	Year	No. of Samples	Positive, *n* (%)	Range (μg/kg)	Mean (μg/kg)	Method	Reference
Argentina	NR ^a^	50	37 (74)	1.4–14	NR	HPLC-FLD ^b^	[42]
Brazil	2002–2003	43	29 (67)	0.1–33.9	NR	HPLC-FLD	[47]
Canada	1998–2000	153	108 (71)	<0.1–26.6	2.74	HPLC-FLD	[48]
China	2012	56	33 (59)	<0.07–12.83	0.99	HPLC-FLD	[49]
Cyprus	2004–2013	43	30 (70)	0.2–13.7	2.2	HPLC-FLD	[50]
Czech Republic	1999–2002	48	18 (38)	1.6–63.6	11.5	HPTLC ^c^	[51]
Czech Republic	NR	12	5 (42)	0.10–2.17	0.46	HPLC-FLD	[52]
Greece	1998–2000	81	60 (74)	0.6–13.8	2.6	HPLC-FLD	[53]
Greece	2011	41	30 (73)	0.1–98.2	NR	HPLC-FLD	[31]
Greece	2012	26	26 (100)	2.8–138.3	47.2	HPLC-FLD/ELISA ^d^	[54]
Hungary	NR	20	18 (90)	0.13–6.2	NR	ELISA	[55]
Iran	2009–2011	40	4 (10)	0.8–4.9	2.41	HPLC-FLD	[56]
Iran	2012–2013	66	39 (59)	<0.16–8.40	2.98	HPLC-FLD	[57]
Iran	2011	38	17 (45)	2.9–18.2	7.0	ELISA	[58]
Italy	NR	35	18 (51)	0.05–12.61	2.6	HPLC-FLD	[59]
Japan	2004–2005	11	7 (64)	0.18–12.5	1.54	HPLC-FLD	[60]
Morocco	2005	20	6 (30)	0.05–4.95	0.96	HPLC-FLD	[61]
Pakistan	2012–2014	170	122 (72)	0.4–12.75	2.10	HPLC-FLD	[62]
Pakistan	2016–2017	17	4 (24)	0.18–18.5	5.6	HPLC-FLD	[63]
Poland	2010–2014	36	17 (47)	1.1–34	6.2	HPLC-FLD	[64]
Poland	NR	5	2 (40)	0.44–1.27	0.86	LC-MS/MS ^e^	[65]
Slovakia	2016	20	15 (75)	0.8–10.6	NR	ELISA	[66]
Spain	NR	3	1 (33)	4.9	4.9	LC-MS/MS	[34]
Sweden	1999–2002	118	96 (84)	<0.1–34.6	NR	HPLC-FLD	[67]
Turkey	NR	20	20 (100)	0.48–8.92	3.80	HPLC-FLD	[68]
Turkey	1998–2004	264	179 (67.8)	0.03–54	3.4	HPLC-FLD	[69]
Turkey	1999–2003	1885	1713 (91)	<0.3–100	1.36	HPLC-FLD	[70]
Turkey	NR	40	26 (65)	0.38–20.90	3.43	HPLC-FLD	[71]
Turkey	NR	53	28 (53)	0.51–58.04	NR	HPLC-FLD	[72]
Turkey	2008–2009	50	4 (8)	0.19–2.59	1.15	HPLC-FLD	[73]
Turkey	NR	60	11 (18)	0.22–5.26	1.61	HPLC-FLD	[74]
Turkey	2015–2016	50	21 (42)	0.14–3.87	0.64	HPLC-FLD	[75]
United Kingdom	NR	60	53 (88)	0.3–53.6	6.4	HPLC-FLD	[76]
United States	2012–2014	109	48 (44)	0.28–15.34	2.26	HPLC-FLD	[43]

^a^ NR: not reported. ^b^ HPLC: high-performance liquid chromatography coupled with fluorescence detection. ^c^ HPTLC: high-performance thin-layer chromatography. ^d^ ELISA: enzyme-linked immunosorbent assay. ^e^ LC-MS/MS: liquid chromatography–tandem mass spectrometry.

**Table 2:** Occurrence of AFs in dried figs marketed in various countries within the last decade.

Country	No. of Samples	Positive, *n* (%)	Range (μg/kg)	Mean (μg/kg)	Method	Reference
Algeria	33	25 (76)	0.22–83.40	NR ^a^	HPLC-FLD ^b^	[90]
China	20	3 (20)	1.80–384.10	129.5	LC-MS/MS ^c^	[91]
Iran	22	13 (59)	0.30–7.00	2.60	HPLC-FLD	[92]
Italy	55	10 (18)	0.19–8.41	NR	HPLC-FLD	[93]
Pakistan	14	4 (29)	NR	3.40	HPLC-FLD	[63]
Turkey	48	11 (23)	0.10–696.30	113.7	HPLC-FLD	[94]
Turkey	130	16 (12)	0.10–12.50	2.66	HPLC-FLD	[95]
Turkey	23,547	2510 (11)	0.20–431.4	5.56	HPLC-FLD	[96]
Turkey	1973	310 (16)	0.59–69.90	5.70	HPLC-FLD	[97]

^a^ NR: not reported. ^b^ HPLC: high-performance liquid chromatography coupled with fluorescence detection. ^c^ LC-MS/MS: liquid chromatography–tandem mass spectrometry.

**Table 3:** Occurrence of AFs and OTA in dates marketed in various countries.

Country	Mycotoxin	No. of Samples	Positive, *n* (%)	Range (μg/kg)	Mean (μg/kg)	Method	Reference
Brazil	OTA	10	2 (20)	0.1–5	NR ^a^	HPLC-FLD ^b^	[47]
China	OTA	40	9 (23)	^c^ LOD–61.4	NR	LC-MS/MS ^d^	[100]
Egypt	AFB_1_	28	1 (4)	14.4	14.4	LC-MS/MS	[32]
Egypt	OTA	28	3 (11)	1.48–6070	NR	LC-MS/MS	[32]
Iran	OTA	10	2 (20)	1.4–3.6	2.5	ELISA ^e^	[58]
Iran	AFs	22	9 (41)	0.9–8.1	2.6	HPLC-FLD	[92]
Iran	OTA	22	5 (23)	0.5–2.1	1.2	HPLC-FLD	[92]
Pakistan	AFs	17	5 (30)	LOD–15.50	3.90	HPLC-FLD	[63]
Pakistan	AFs	8	2 (25)	2.1–2.9	2.5	HPLC-FLD	[80]
Pakistan	AFs	15	9 (60)	LOD–18.8	6.32	HPLC-FLD	[81]
Pakistan	AFs	96	38 (40)	LOD-26.6	4.11	HPLC-FLD	[101]
Pakistan	AFs	170	25 (15)	0.24–5.87	NR	HPLC-FLD	[102]
Tunisia	OTA	48	18 (38)	0.57–3.34	1.26	LC-MS/MS	[32]
Yemen	AFs	20	2 (10)	110–180	145	TLC ^f^	[103]

^a^ NR: not reported. ^b^ HPLC: high-performance liquid chromatography coupled with fluorescence detection. ^c^ LOD: limit of detection. ^d^ LC-MS/MS: liquid chromatography–tandem mass spectrometry. ^e^ ELISA: enzyme-linked immunosorbent assay. ^f^ TLC: thin-layer chromatography.

**Table 4:** Occurrence of AFs and OTA in prunes marketed in various countries.

Country	Mycotoxin	No. of Samples	Positive, *n* (%)	Range (μg/kg)	Mean (μg/kg)	Method	Reference
Brazil	OTA	21	1 (5)	<5	<5	HPLC-FLD ^a^	[47]
Egypt	OTA	3	3 (100)	210–280	NR ^b^	TLC ^c^	[109]
Iran	AFB_1_	15	2 (13)	0.23–1.17	0.70	HPLC-FLD	[110]
Iran	OTA	15	3 (20)	0.22–2.62	1.28	HPLC-FLD	[110]
Pakistan	AFs	16	6 (38)	^d^ LOD–8.5	3.80	HPLC-FLD	[63]
Pakistan	AFs	21	8 (38)	0.04–14.76	3.72	HPLC-FLD	[81]
Pakistan	AFs	10	3 (30)	2.36–7.41	1.31	HPLC-FLD	[102]

^a^ HPLC: high-performance liquid chromatography coupled with fluorescence detection. ^b^ NR: not reported. ^c^ TLC: thin-layer chromatography. ^d^ LOD: limit of detection.

**Table 5:** Occurrence of AFs and OTA in dried apricots marketed in various countries.

Country	Mycotoxin	No. of Samples	Positive, *n* (%)	Range (μg/kg)	Mean (μg/kg)	Method	Reference
Iran	OTA	15	1 (7)	2.8	2.8	ELISA ^a^	[58]
Iran	AFs	22	18 (82)	0.4–8.5	2.9	HPLC-FLD ^b^	[92]
Iran	AFB_1_	30	9 (30)	0.21–5.33	0.88	HPLC-FLD	[110]
Iran	OTA	30	1 (3)	2.83	2.83	HPLC-FLD	[110]
Pakistan	AFs	13	3 (23)	^c^ LOD–11.85	4.80	HPLC-FLD	[63]
Pakistan	AFs	20	4 (20)	1.5–10.3	4.55	HPLC-FLD	[80]
Pakistan	AFs	20	7 (35)	LOD–11.5	4.75	HPLC-FLD	[81]
Pakistan	AFs	65	21 (32)	0.31–11.1	1.02	HPLC-FLD	[102]
Turkey	AFs	26	2 (8)	LOD–10.5	0.61	ELISA	[30]
Turkey	OTA	26	6 (23)	LOD–34.4	6.1	ELISA	[30]
Turkey	OTA	20	1 (5)	0.97	0.97	HPLC-FLD	[72]
Turkey	AFB_1_	15	3 (20)	NR ^d^	1.44	ELISA	[113]

^a^ ELISA: enzyme-linked immunosorbent assay. ^b^ HPLC: high-performance liquid chromatography coupled with fluorescence detection. ^c^ LOD: limit of detection. ^d^ NR: not reported.

## Data Availability

No new data were created.
